# Atrial Fibrillation Is a Major Risk Factor for Stroke, Especially in Women: The Jichi Medical School Cohort Study

**DOI:** 10.2188/jea.JE20090149

**Published:** 2011-03-05

**Authors:** Hiroyuki Iwahana, Shizukiyo Ishikawa, Joji Ishikawa, Tomoyuki Kabutoya, Kazunori Kayaba, Tadao Gotoh, Eiji Kajii

**Affiliations:** 1 Department of Internal Medicine, Kamiichi General Hospital, Toyama, Japan; 2 Division of Community and Family Medicine, Jichi Medical University, Tochigi, Japan; 3 Division of Cardiovascular Medicine, Jichi Medical University, Tochigi, Japan; 4 Chichibu Municipal Hospital, Saitama, Japan; 5 School of Health and Social Services, Saitama Prefectural University, Saitama, Japan; 6 Wara National Health Insurance Hospital, Gifu, Japan

**Keywords:** atrial fibrillation, stroke, women, cohort study

## Abstract

**Background:**

Only a few population-based cohort studies have investigated the impact of atrial fibrillation (AF) on stroke in Japan.

**Methods:**

A total of 10 929 participants (4147 men and 6782 women) were included in this population-based prospective cohort study. Baseline data, including electrocardiograms (ECGs) to ascertain AF status, were obtained from April 1992 through July 1995 in 12 areas in Japan. Cox proportional hazards models were used to analyze the association of AF with stroke.

**Results:**

A total of 54 participants had AF (0.49%). The mean follow-up period was 10.7 years, during which 405 strokes were identified; 12 of these occurred in participants with AF. The crude incidence of stroke in participants with and without AF was 14.9 and 4.5 per 1000 person-years in men, respectively, and 39.3 and 2.7 per 1000 person-years in women. After adjusting for geographical area, sex, age, smoking status, drinking status, obesity, hypertension, dyslipidemia, and diabetes mellitus, the hazard ratios (95% confidence interval) of AF in all participants and in male and female participants were 4.11 (2.28–7.41), 2.12 (0.77–5.84), and 10.6 (5.01–22.4), respectively. The population attributable fraction (PAF) of stroke caused by AF was 2.2%; the PAFs were 1.0% and 3.6% in men and women, respectively.

**Conclusions:**

The present Japanese population-based prospective cohort study showed that AF is a major risk factor for stroke, especially in women.

## INTRODUCTION

Atrial fibrillation (AF) is the most common sustained arrhythmia and is a major risk factor for stroke.^1^^,^^2^ The prevalence of AF is rising with the increasing age of many populations,^1^^–^^8^ and it is more frequent in men than in women.^2^^,^^5^^–^^8^ Studies have shown that the risk of stroke is 2 to 7 times higher in people with AF as compared with those without AF.^3^^,^^4^^,^^9^^–^^11^ It has been suggested that the risk of stroke due to AF is higher in women than in men.^11^^–^^15^ AF contributes to a number of medical, social, and economic problems by increasing the burdens on outpatient clinics, the extent of pharmacological treatment, admissions to hospital, and the incidence of disability due to cardiovascular diseases.^13^^,^^16^

In Japan, the percentage of the population in older age groups is increasing at the highest rate in the world. The estimated number of persons with AF is also rising rapidly in Japan.^5^ However, there have only been a few Japanese population-based studies of the effect of AF on stroke.^17^^–^^20^ Tanaka et al^17^ and Tanizaki et al^19^ conducted an epidemiologic study of cerebral infarction as a stroke subtype. Ohsawa et al^20^ examined mortality risk, including stroke death, attributable to AF. In the Shibata study, Nakayama et al^18^ classified stroke into 4 subtypes: hemorrhagic stroke, ischemic stroke, subarachnoid hemorrhage (SAH), and undetermined strokes. However, none of these studies found an effect of AF on hemorrhagic stroke or SAH. Nor did they address the differing effects of AF on stroke incidence in men and women. In this study, data from the JMS cohort were used to estimate hazard ratios (HRs) of stroke associated with AF, after adjusting for geographical area, sex, age, smoking status, drinking status, obesity, hypertension, dyslipidemia, and diabetes mellitus using Cox proportional hazards models. We also analyzed the effect of AF on all strokes, and on hemorrhagic stroke, ischemic stroke, and SAH, in both men and women. To estimate the proportion of strokes due to AF in this population, we calculated population attributable fractions (PAFs) of AF for all strokes and for stroke subtypes.

## METHODS

The Jichi Medical School (JMS) Cohort Study is a population-based prospective cohort study. Its primary objective was to clarify the relationship between potential risk factors and health outcomes such as stroke, cardiovascular disease, and cancer in 12 local communities across Japan. The baseline data of this cohort study were obtained between April 1992 and July 1995. A detailed description of standardized data collection at baseline has been previously published.^21^^,^^22^ The study design and procedure were approved by the Ethical Committee at Jichi Medical University.

### Participants

Invitations to this mass screening were issued by local government offices in each community, and personal invitations were also sent to all potential participants by mail. The age of the adults participating in the mass screening examinations was 40 to 69 years in 8 communities, 20 to 69 years in 1 community, and 35 years or older (ie, no upper age limit) in 1 community; there was no age limit in 2 communities. The overall participation rate for those invited to the mass screening examination program was 65.4%. Written informed consent to participate in the study was obtained individually from all respondents to the mass screening.

In total, 12 490 people were available for participation. However, 95 declined follow-up, and 7 could not be contacted after collection of baseline data. We also excluded 109 individuals with a history of stroke, 1347 with missing electrocardiogram (ECG) information, and 3 with both a past history of stroke and missing ECG data. Thus, the final number of study participants was 10 929 (4147 men and 6782 women).

### Initial survey and definition of status

At the baseline examination, each participant filled out a questionnaire on their lifestyle and medical history. A series of physical examinations was performed, and a 12-lead ECG at rest was recorded. A diagnosis of AF was based on the independent determination of 2 cardiologists, who reviewed a single baseline ECG. In the event of a disagreement, the final decision was made after deliberation by the approval committee. Smoking status was classified as smoker or nonsmoker. Drinking status was classified as drinker and nondrinker. Body mass index (BMI) was calculated as weight in kilograms divided by the subject’s height in meters squared (kg/m^2^). Obesity was defined as a BMI of 25 kg/m^2^ or higher. Blood pressure was measured once with a fully automated sphygmomanometer, the BP203RV-II (Nippon Colin, Komaki, Japan), that was placed on the right arm of a seated participant who had rested in a sitting position for at least 5 minutes before the measurement. Hypertension was defined as a systolic blood pressure of 140 mm Hg or higher, a diastolic blood pressure of 90 mm Hg or higher, or current use of antihypertensive agents. Dyslipidemia was defined as a total cholesterol level of 220 mg/dl or higher, high-density lipoprotein cholesterol lower than 40 mg/dl, or current use of medication for hyperlipidemia. Diabetes mellitus was defined as fasting blood glucose level of 126 mg/dl or higher, casual blood glucose of 200 mg/dl or higher, or current use of diabetes medication.

### Follow-up study

After enrolling in the study, participants were asked at the annual mass screening whether they had developed stroke or cardiovascular disease. Participants who did not attend the annual mass screening examination were contacted by mail or phone. Medical records were checked, and if an incident case was found, we requested duplicate films from computer tomography or magnetic resonance for stroke, or ECGs for myocardial infarction. Data on participants who left the study due to moving out of the area were obtained annually from each municipal government.

Stroke and myocardial infarction were diagnosed as described previously.^21^^,^^22^ A diagnosis committee—composed of 1 radiologist, 1 neurologist, and 2 cardiologists—diagnosed stroke and myocardial infarction independently from the data collection groups. Stroke was defined as a focal and nonconvulsive neurological deficit of sudden onset persisting at least 24 hours. Stroke subtypes were confirmed based on computed tomography or magnetic resonance imaging.^23^ A diagnosis of myocardial infarction was made using the criteria from the World Health Organization’s MONICA project.^24^

### Statistical analysis

The baseline data were classified by AF status and sex. Ages were compared using the unpaired *t*-test. Continuous variables were expressed as age-adjusted means with 95% confidence intervals (CIs) and compared by analysis of covariance (ANCOVA). Data for proportions were expressed as percentages, which were compared using the chi-square test. Using the baseline data, the incidence of AF was calculated by sex and age group. The follow-up period was defined as the period from the date of baseline collection to either the date of incidence (stroke, myocardial infarction, or death), the end of follow-up in the respective area, or the date of moving out of the study area. Incidence rates were calculated and expressed per 1000 person-years. Cumulative stroke incidence was estimated using the Kaplan-Meier method and *P*-values were calculated using the log-rank (Mantel–Cox) method. The Cox proportional hazards model was used to estimate HRs for stroke, adjusting for geographical area, age, sex, smoking status, drinking status, obesity, hypertension, dyslipidemia, diabetes mellitus and AF. Population attributable fraction (PAF) was calculated as Pe × (HR − 1)/HR, in which Pe is the proportion of stroke cases exposed to the risk factor for each type of stroke or for all stroke cases.^25^^,^^26^ The analyses were performed using SPSS 16.0J for Windows and Microsoft Office Excel 2003.

## RESULTS

The baseline characteristics of the participants are shown in Table 1. Although mean age was significantly higher in participants with AF than in those without AF, there was no significant difference in mean age with respect to AF status in men or women. BMI and high-density lipoprotein cholesterol were significantly higher in AF participants than in non-AF participants in men and women, respectively. In non-AF participants, systolic blood pressure, diastolic blood pressure, triglyceride, and blood sugar were significantly higher in men, whereas, BMI, total cholesterol, and high-density lipoprotein cholesterol were significantly higher in women. In both the AF and non-AF groups, significantly more men than women were smokers and drinkers.

**Table 1.** Baseline data from JMS cohort study, by atrial fibrillation (AF) status and sex

	Without AF					With AF				
		
	Men		Women			Men		Women		
		
No.	4117		6758			30		24		

Characteristic	Mean	95% CI	Mean	95% CI	*P*	Mean	95% CI	Mean	95% CI	*P*
Age (yrs)	55.5	55.1–55.8	55.6	55.4–55.9	0.403	64.9***	60.8–69.1	66.8***	62.4–71.2	0.346
BMI	23.0	22.9–23.1	23.2	23.1–23.3	<0.001	24.0**	23.1–24.8	24.2	23.3–25.0	0.805
SBP (mm Hg)	134.0	133.3–134.6	130.7	130.2–131.2	<0.001	127.9	122.5–133.3	124.6	119.2–130.0	0.813
DBP (mm Hg)	80.2	79.8–80.6	77.3	77.0–77.6	<0.001	77.6	74.4–80.8	74.7	71.5–77.9	0.684
T-CHOL (mg/dl)	187.2	186.2–188.3	199.6	198.7–200.4	<0.001	180.9	171.8–189.9	193.2	184.1–202.3	0.135
TG (mg/dl)	128.7	126.3–131.1	110.5	108.6–112.4	<0.001	112.5	92.2–132.7	94.3	74.0–114.6	0.287
HDL-C (mg/dl)	48.8	48.3–49.2	52.4	52.1–52.7	<0.001	53.6	50.1–57.0	57.2*	53.8–60.7	0.322
BS (mg/dl)	107.6	106.8–108.5	102.1	101.5–102.8	<0.001	107.4	100.3–114.4	101.8	94.8–108.9	0.541

Smoking status (%)					<0.001					<0.001
Smoker	49.9		5.7			41.4		4.3		
Nonsmoker	50.1		94.3			58.6		95.7		
Drinking status (%)					<0.001					<0.001
Drinker	75.0		24.4			74.6		22.3		
Nondrinker	25.0		75.6			25.4		77.7		

Ages were compared using the unpaired *t* test.

On ANCOVA, continuous values except for age were adjusted using age 60 years.

The chi-square test was used for comparisons of proportions.

*P < 0.05 for comparison of participants with and without AF.

**P < 0.01 for comparison of participants with and without AF.

***P < 0.001 for comparison of participants with and without AF.

95% CI: 95% confidence interval, BMI: body mass index, SBP: systolic blood pressure, DBP: diastolic blood pressure, T-CHOL: total cholesterol, TG: triglyceride, HDL-C: high-density lipoprotein cholesterol, BS: blood sugar.

As shown in Table 2, there were 54 participants with AF (0.49%). The prevalence of AF increased with age in both men and women. In men, the prevalence of AF increased at age 50 to 59 years and progressively increased thereafter. In women, the prevalence of AF drastically increased and, at age 70 years or older, surpassed that in men. However, the proportion of men with AF (0.72%) was higher than in women (0.35%; *P* = 0.008).

**Table 2.** Prevalence of atrial fibrillation by age group and sex

Age, yrs	Men		Women		Total	
		
	AF(+)/*n*	%	AF(+)/*n*	%	AF(+)/*n*	%
–39	0/367	0	0/513	0	0/880	0
40–49	0/897	0	1/1376	0.07	1/2273	0
50–59	5/995	0.50	2/1974	0.10	7/2969	0.24
60–69	21/1651	1.27	11/2595	0.42	32/4246	0.75
70+	4/237	1.69	10/324	3.09	14/561	2.50
Total	30/4147	0.72	24/6782	0.35	54/10 929	0.49

AF: atrial fibrillation.

The mean duration of follow-up was 10.7 years. During the follow-up period, we identified 405 strokes, including 91 hemorrhagic strokes, 262 ischemic strokes, 51 SAHs, and 1 unspecified stroke. Therefore, with hemorrhagic stroke as the reference (hemorrhagic stroke:ischemic stroke:SAH:unspecified stroke), the ratio for each stroke subtype was 1:2.88:0.56:0.01.

The cumulative stroke incidences for participants with and without AF are shown in the Figure. Log-rank analysis revealed that the cumulative stroke incidence was higher in AF participants than in non-AF participants in both men (*P* = 0.014) and women (*P* < 0.001). Cox proportional hazards models were used to analyze the associations of all strokes with various risk factors (Table 3), including geographical area, sex, age, smoking status, drinking status, obesity, hypertension, dyslipidemia, diabetes mellitus, and AF. Stroke was associated with male sex, smoking status, hypertension, diabetes mellitus, and AF, which was the strongest risk factor for stroke (Table 3).

**Table 3.** Cox hazard ratios and 95% confidence intervals for overall stroke risk associated with various risk factors

Characteristic	HR	95% CI
Male sex	1.39	1.06–1.81
Age, per 10-year increment	1.09	1.07–1.10
Smoking	1.34	1.03–1.76
Drinking	1.03	0.81–1.31
Obesity	0.96	0.75–1.23
Hypertension	2.65	2.11–3.31
Dyslipidemia	1.08	0.87–1.35
Diabetes mellitus	2.07	1.40–3.06
AF	4.11	2.28–7.41

Cox hazard ratios were also adjusted by geographical area.

HR: hazard ratio, 95% CI: 95% confidence interval, AF: atrial fibrillation.

As shown in Table 4, there were 198 and 4 strokes in men without and with AF, respectively. The crude incidence rates were 4.5 and 14.9 per 1000 person-years; thus, the crude incidence rate in AF participants was about 3 times that of non-AF participants. In women, there were 195 and 8 strokes, corresponding to crude incidence rates of 2.7 and 39.3 per 1000 person-years, in non-AF and AF participants, respectively. The crude incidence rate in AF participants was about 15 times that of non-AF participants.

**Table 4.** Number of incident strokes, crude incidence rates, multivariate-adjusted hazard ratios, and population attributable fractions for atrial fibrillation (AF) by stroke subtype

	No.	Crude incidence^a^	No.	Crude incidence^a^	HR (95% CI)^b^	PAF (%)
Total	Without AF (*n* = 10 875)		With AF (*n* = 54)			
Hemorrhagic stroke	89	0.8	2	3.7	2.90 (0.69–12.2)	1.4
Ischemic stroke	253	2.2	9	16.7	4.51 (2.28–8.94)	2.7
SAH	50	0.4	1	1.9	4.09 (0.54–30.7)	1.5
All strokes	393	3.4	12	22.2	4.11 (2.28–7.41)	2.2

Men	Without AF (*n* = 4117)		With AF (*n* = 30)			
Hemorrhagic stroke	42	1.0	1	3.7	3.15 (0.40–25.0)	1.6
Ischemic stroke	143	3.3	3	11.1	2.16 (0.67–6.97)	1.1
SAH	13	0.3	0	0	0	—
All strokes	198	4.5	4	14.9	2.12 (0.77–5.84)	1.0

Women	Without AF (*n* = 6758)		With AF (*n* = 24)			
Hemorrhagic stroke	47	0.6	1	4.9	5.93 (0.77–45.6)	1.7
Ischemic stroke	110	1.5	6	29.5	13.2 (5.43–32.1)	4.8
SAH	37	0.5	1	4.9	8.69 (1.10–68.4)	2.3
All strokes	195	2.7	8	39.3	10.6 (5.01–22.4)	3.6

HR: hazard ratio, 95% CI: 95% confidence interval, PAF: population attributable fraction, SAH: subarachnoid hemorrhage.

^a^per 1000 person-years.

^b^HRs were calculated using a Cox proportional hazard model adjusted for geographical area, sex, age, smoking status, drinking status, obesity, hypertension, dyslipidemia, and diabetes mellitus.

The HRs for stroke associated with AF were estimated using a Cox proportional hazards model (Table 4). AF increased the risk of stroke by factors of 2 and 11 in men and women, respectively. Thus, the effect of AF on stroke in women was about 5 times greater than in men. Strokes were divided by subtype into hemorrhagic stroke, ischemic stroke, and SAH (Table 4). In men, AF increased the risks of hemorrhagic stroke and ischemic stroke by factors of approximately 3 and 2, respectively. In women, AF increased the risks of hemorrhagic stroke, ischemic stroke, and SAH by factors of approximately 6, 13, and 9, respectively. The increases in overall stroke risk associated with AF was statistically significant in women, but not in men.

As shown in Table 4, with respect to PAF, AF contributed to only 1.4% of hemorrhagic stroke incidence. The PAF for ischemic stroke was 1.1% in men and 4.8% in women. For all strokes, the PAFs were 1.0% and 3.6% in men and women, respectively. Thus, the PAFs for ischemic stroke and all strokes in women were approximately 4 times those of men.

## DISCUSSION

**Figure 1.**
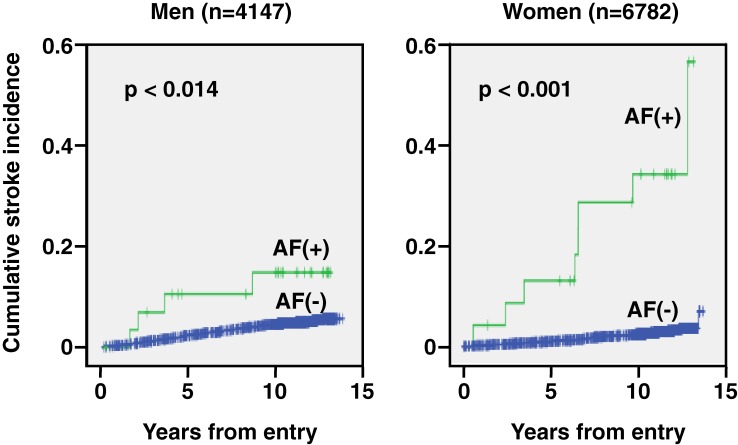
Cumulative stroke incidence by sex and atrial fibrillation (AF) status. *P* values were calculated using the log-rank (Mantel–Cox) method.

The JMS Cohort Study is a prospective population-based cohort study of risk factors for cardiovascular disease in Japan.^21^^,^^22^ There are few population-based studies of the effect of AF on stroke in Japan. Among them, the number of participants in the present study is 1.3, 5, and 7 times those of the NIPPON DATA80,^20^ Shibata Study,^17^^,^^18^ and Hisayama Study,^19^ respectively. The present study showed that the prevalence of AF was higher in men than in women and that the prevalence of AF increased with age in both men and women (Table 2). In addition, cumulative stroke incidences were significantly higher in AF participants than in non-AF participants in both men and women (Figure 1). Male sex, smoking status, hypertension, diabetes mellitus, and AF were shown to be major risk factors for stroke, after adjusting for geographical area, sex, age, smoking status, drinking status, obesity, hypertension, dyslipidemia, diabetes mellitus, and AF (Table 3), which was the strongest risk factor. AF also had the greatest effect on ischemic stroke and was a more unambiguous risk factor for stroke in women than in men (Table 4).

In the Japanese general population, the prevalence of AF was reported to range between 0.56% and 1.6%.^5^^–^^8^ The prevalence of AF in Western countries is higher than in Japan,^1^^–^^4^ and apparently increases with age.^1^^–^^8^ AF is also more common in men than in women.^2^^,^^5^^–^^8^ As shown in Table 2, the prevalence of AF in the present study was 0.49%, which was somewhat lower than in previous reports. This may be because the participants in the present study were healthier, as they were regular participants in annual mass screening examinations, and because we excluded participants aged 70 years or older in 9 of 12 areas. Thus, selection bias might have affected the results. However, our results confirmed that the prevalence of AF increases with age and that AF is more common in men than in women (Table 2).

In the JMS cohort study, the ratio of each stroke subtype (hemorrhagic stroke:ischemic stroke:SAH:unspecified stroke) was 1:2.88:0.56:0.01. In the Takasima Stroke Registry,^27^ the equivalent ratio was 1:3.10:0.44:0.07, which is quite similar to our results. However, in the Framingham study,^14^ the ratio of hemorrhagic stroke:ischemic stroke:SAH:others was 1:13.70:1.44:0.52. Therefore, the ratio of ischemic stroke was substantially higher in the United States than in Japan. Most studies in East Asia, including those in Japan, have suggested that the proportion of hemorrhagic stroke in those populations is significantly higher than in whites.^28^

In both men and women, cumulative stroke incidence was higher in participants with AF than in those without the condition (Figure 1). As shown in Table 3, AF was an independent risk factor for stroke after adjusting for geographical area, sex, age, smoking status, drinking status, obesity, hypertension, dyslipidemia, and diabetes mellitus; AF quadrupled the risk of stroke. Previous studies in Japan reported that AF increased the risk of stroke, including cerebral infarction and stroke death, by a factor of 3 to 7.^17^^–^^20^ However, none of those studies investigated the effect of AF on stroke subtypes. In the present study, we classified stroke as hemorrhagic stroke, ischemic stroke, and SAH, and analyzed the influence of AF on each of these subtypes (Table 4). Although AF increased the risk for each type of stroke, the increase was not statistically significant for hemorrhagic stroke in either men or women, or for ischemic stroke and SAH in men. If a greater number of participants had been enrolled, the increase in risk might have been significant. Therefore, to clarify the effect of AF on hemorrhagic stroke and SAH, a longer observation period or a meta-analysis will be necessary.

The effect of AF on stroke was analyzed using Cox proportional hazards models (Table 4). The HRs for all types of stroke except hemorrhagic stroke were significant in women, but not in men. It is particularly noteworthy that the effect of AF on the risk of stroke in women was about 5 times that in men (Table 4). With respect to PAF, in women, the contribution of AF to ischemic stroke and overall stroke risk was approximately 4 times that in men (Table 4). In the present study, the highest PAF was only 4.8% because of the low prevalence of AF. However, the prevalence of AF increases with age,^1^^–^^8^ and Japan is one of the most rapidly aging countries in the world.^29^ Therefore, the PAF of AF is expected to increase, and AF will likely have a greater impact on Japanese society in the near future. Friberg et al^15^ reported that AF is a much more significant risk factor for stroke in women than in men. Some reports also showed that women with AF were more likely to have a stroke than men with AF.^11^^–^^14^ In addition, women with stroke were more likely to have AF than men with stroke.^30^^–^^32^ However, it is not known why AF is associated with a higher risk of stroke in women.

This study has some limitations that warrant mention. As shown in Table 4, the HRs for all types of stroke were not significant in men, and the HR for hemorrhagic stroke was not significant in women. The number of participants with AF was only 54; therefore, the statistical power of the study may have been insufficient. Second, the prevalence of AF increases with age, but because participants aged 70 years or older were excluded in 9 of the 12 study areas, the number of such participants was small (Table 2). This might be a form of selection bias that led to the result indicating that the prevalence of AF at age 70 or older was higher in women than in men, which contradicts the results of previous reports. However, despite the lack of statistical power and possibility of selection bias, any distortion of relative risk estimates is likely to be small. A third limitation was that a diagnosis of AF was based on a single, baseline ECG recording. Therefore, AF could not differentiated as paroxysmal, persistent, or permanent. However, the risks for stroke and non-central nervous system embolism were reported to be similar in individuals with paroxysmal AF and those with sustained AF.^33^ In the present study, participants with paroxysmal AF were probably not classified as having AF, and some participants probably developed AF during the follow-up period, so the effect of AF on stroke might have been underestimated. Another limitation of the study was that the anticoagulant agent warfarin apparently decreases the risk of stroke in patients with AF.^1^^,^^11^^,^^34^ Anticoagulation therapy for stroke prevention in AF participants may increase hemorrhagic risk, thus possibly increasing the risk for hemorrhagic stroke or SAH. This study showed that AF may increase the risk of hemorrhagic stroke and SAH (Table 4). However, we did not collect data concerning the use of anticoagulant agents. Therefore, in future studies of AF and stroke, anticoagulant therapy must be evaluated as a confounding factor.

AF is a major risk factor for stroke, particularly in women, and its prevalence increases with age. The proportion of elderly people is increasing rapidly in Japan, and AF has recently been treated with anticoagulant agents to prevent cardiovascular disease. Therefore, the importance of AF as a medical, economic, and social issue is continuing to increase.

## References

[1] Feinberg WM , Blackshear JL , Laupacis A , Kronmal R , Hart RG Prevalence, age distribution, and gender of patients with atrial fibrillation. Analysis and implications . Arch Intern Med. 1995;155:469–73 10.1001/archinte.155.5.469 7864703

[2] Go AS , Hylek EM , Phillips KA , Chang Y , Henault LE , Selby JV , Prevalence of diagnosed atrial fibrillation in adults: national implications for rhythm management and stroke prevention: the AnTicoagulation and Risk Factors in Atrial Fibrillation (ATRIA) Study . JAMA. 2001;285:2370–5 10.1001/jama.285.18.2370 11343485

[3] Wolf PA , Abbott RD , Kannel WB Atrial fibrillation: a major contributor to stroke in the elderly. The Framingham Study . Arch Intern Med. 1987;147:1561–4 10.1001/archinte.147.9.1561 3632164

[4] Krahn AD , Manfreda J , Tate RB , Mathewson FA , Cuddy TE The natural history of atrial fibrillation: incidence, risk factors, and prognosis in the Manitoba Follow-Up Study . Am J Med. 1995;98:476–84 10.1016/S0002-9343(99)80348-9 7733127

[5] Ohsawa M , Okayama A , Sakata K , Kato K , Itai K , Onoda T , Rapid increase in estimated number of persons with atrial fibrillation in Japan: an analysis from national surveys on cardiovascular diseases in 1980, 1990 and 2000 . J Epidemiol. 2005;15:194–6 10.2188/jea.15.194 16195640 PMC7904304

[6] Iguchi Y , Kimura K , Aoki J , Kobayashi K , Terasawa Y , Sakai K , Prevalence of atrial fibrillation in community-dwelling Japanese aged 40 years or older in Japan: analysis of 41,436 non-employee residents in Kurashiki-city . Circ J. 2008;72:909–13 10.1253/circj.72.909 18503215

[7] Ohsawa M , Itai K , Tanno K , Onoda T , Ogawa A , Nakamura M , Cardiovascular risk factors in the Japanese northeastern rural population . Int J Cardiol. 2009;137:226–35 10.1016/j.ijcard.2008.06.052 18707775

[8] Inoue H , Fujiki A , Origasa H , Ogawa S , Okumura K , Kubota I , Prevalence of atrial fibrillation in the general population of Japan: an analysis based on periodic health examination . Int J Cardiol. 2009;137:102–7 10.1016/j.ijcard.2008.06.029 18691774

[9] Wolf PA , Dawber TR , Thomas HE Jr , Kannel WB Epidemiologic assessment of chronic atrial fibrillation and risk of stroke: the Framingham Study . Neurology. 1978;28:973–7 570666 10.1212/wnl.28.10.973

[10] Flegel KM , Shipley MJ , Rose G Risk of stroke in non-rheumatic atrial fibrillation . Lancet. 1987;1(8532):526–9 10.1016/S0140-6736(87)90174-7 2881082

[11] Wolf PA , Abbott RD , Kannel WB Atrial fibrillation as an independent risk factor for stroke: the Framingham Study . Stroke. 1991;22:983–8 1866765 10.1161/01.str.22.8.983

[12] Boysen G , Nyboe J , Appleyard M , Sorensen PS , Boas J , Somnier F , Stroke incidence and risk factors for stroke in Copenhagen, Denmark . Stroke. 1988;19:1345–53 3188119 10.1161/01.str.19.11.1345

[13] Wolf PA , Mitchell JB , Baker CS , Kannel WB , D’Agostino RB Impact of atrial fibrillation on mortality, stroke, and medical costs . Arch Intern Med. 1998;158:229–34 10.1001/archinte.158.3.229 9472202

[14] Wang TJ , Massaro JM , Levy D , Vasan RS , Wolf PA , D’Agostino RB , A risk score for predicting stroke or death in individuals with new-onset atrial fibrillation in the community: the Framingham Heart Study . JAMA. 2003;290:1049–56 10.1001/jama.290.8.1049 12941677

[15] Friberg J , Scharling H , Gadsbøll N , Truelsen T , Jensen GB ; Copenhagen City Heart Study Comparison of the impact of atrial fibrillation on the risk of stroke and cardiovascular death in women versus men (The Copenhagen City Heart Study) . Am J Cardiol. 2004;94:889–94 10.1016/j.amjcard.2004.06.023 15464671

[16] Ringborg A , Nieuwlaat R , Lindgren P , Jönsson B , Fidan D , Maggioni AP , Costs of atrial fibrillation in five European countries: results from the Euro Heart Survey on atrial fibrillation . Europace. 2008;10:403–11 10.1093/europace/eun048 18326853

[17] Tanaka H , Hayashi M , Date C , Imai K , Asada M , Shoji H , Epidemiologic studies of stroke in Shibata, a Japanese provincial city: preliminary report on risk factors for cerebral infarction . Stroke. 1985;16:773–80 4049440 10.1161/01.str.16.5.773

[18] Nakayama T , Date C , Yokoyama T , Yoshiike N , Yamaguchi M , Tanaka H A 15.5-year follow-up study of stroke in a Japanese provincial city. The Shibata Study . Stroke. 1997;28:45–52 8996487 10.1161/01.str.28.1.45

[19] Tanizaki Y , Kiyohara Y , Kato I , Iwamoto H , Nakayama K , Shinohara N , Incidence and risk factors for subtypes of cerebral infarction in a general population: the Hisayama study . Stroke. 2000;31:2616–22 11062284 10.1161/01.str.31.11.2616

[20] Ohsawa M , Okayama A , Okamura T , Itai K , Nakamura M , Tanno K , Mortality risk attributable to atrial fibrillation in middle-aged and elderly people in the Japanese general population: nineteen-year follow-up in NIPPON DATA80 . Circ J. 2007;71:814–9 10.1253/circj.71.814 17526974

[21] Ishikawa S , Gotoh T , Nago N , Kayaba K ; Jichi Medical School (JMS) Cohort Study Group The Jichi Medical School (JMS) Cohort Study: design, baseline data and standardized mortality ratios . J Epidemiol. 2002;12:408–17 12462275 10.2188/jea.12.408 PMC10681813

[22] Ishikawa S , Kayaba K , Gotoh T , Nago N , Nakamura Y , Tsutsumi A , Incidence of total stroke, stroke subtypes, and myocardial infarction in the Japanese population: the JMS Cohort Study . J Epidemiol. 2008;18:144–50 10.2188/jea.JE2007438 18603825 PMC4771583

[23] Adams HP Jr , Bendixen BH , Kappelle LJ , Biller J , Love BB , Gordon DL , Classification of subtype of acute ischemic stroke. Definitions for use in a multicenter clinical trial. TOAST. Trial of Org 10172 in Acute Stroke Treatment . Stroke. 1993;24:35–41 7678184 10.1161/01.str.24.1.35

[24] The World Health Organization MONICA Project (monitoring trends and determinants in cardiovascular disease): a major international collaboration. WHO MONICA Project Principal Investigators . J Clin Epidemiol. 1988;41:105–14 10.1016/0895-4356(88)90084-4 3335877

[25] Nakayama T , Yokoyama T , Yoshiike N , Zaman MM , Date C , Tanaka H , Population attributable fraction of stroke incidence in middle-aged and elderly people: contributions of hypertension, smoking and atrial fibirillation . Neuroepidemiology. 2000;19:217–26 10.1159/000026259 10859502

[26] Rockhill B , Newman B , Weinberg C Use and misuse of population attributable fractions . Am J Public Health. 1998;88:15–9 10.2105/AJPH.88.1.15 9584027 PMC1508384

[27] Kita Y , Turin TC , Rumana N , Sugihara H , Morita Y , Hirose K , Surveillance and measuring trends in Japan: The Takashima Stroke Registry (1988-present) . Int J Stroke. 2007;2:129–32 10.1111/j.1747-4949.2007.00107.x 18705970

[28] Sudlow CL , Warlow CP Comparable studies of the incidence of stroke and its pathological types: results from an international collaboration. International Stroke Incidence Collaboration . Stroke. 1997;28:491–9 9056601 10.1161/01.str.28.3.491

[29] Population Division, Department of Economic and Social Affairs, United Nations [Internet]. New York: World Population Ageing 2009 [updated 2009 Dec; cited 2010 Oct 12]. Available from: http://www.un.org/esa/population/publications/WPA2009/WPA2009_WorkingPaper.pdf

[30] Di Carlo A , Lamassa M , Baldereschi M , Pracucci G , Basile AM , Wolfe CD , Sex differences in the clinical presentation, resource use, and 3-month outcome of acute stroke in Europe: data from a multicenter multinational hospital-based registry . Stroke. 2003;34:1114–9 10.1161/01.STR.0000068410.07397.D7 12690218

[31] Roquer J , Campello AR , Gomis M Sex differences in first-ever acute stroke . Stroke. 2003;34:1581–5 10.1161/01.STR.0000078562.82918.F6 12805490

[32] Kapral MK , Fang J , Hill MD , Silver F , Richards J , Jaigobin C , Sex differences in stroke care and outcomes: results from the Registry of the Canadian Stroke Network . Stroke. 2005;36:809–14 10.1161/01.STR.0000157662.09551.e5 15731476

[33] Hohnloser SH , Pajitnev D , Pogue J , Healey JS , Pfeffer MA , Yusuf S , Incidence of stroke in paroxysmal versus sustained atrial fibrillation in patients taking oral anticoagulation or combined antiplatelet therapy: an ACTIVE W Substudy . J Am Coll Cardiol. 2007;50:2156–61 10.1016/j.jacc.2007.07.076 18036454

[34] Hylek EM , Go AS , Chang Y , Jensvold NG , Henault LE , Selby JV , Effect of intensity of oral anticoagulation on stroke severity and mortality in atrial fibrillation . N Engl J Med. 2003;349:1019–26 10.1056/NEJMoa022913 12968085

